# A Bacterial Glucanotransferase Can Replace the Complex Maltose Metabolism Required for Starch to Sucrose Conversion in Leaves at Night*

**DOI:** 10.1074/jbc.M113.497867

**Published:** 2013-08-15

**Authors:** Christian Ruzanski, Julia Smirnova, Martin Rejzek, Darrell Cockburn, Henriette L. Pedersen, Marilyn Pike, William G. T. Willats, Birte Svensson, Martin Steup, Oliver Ebenhöh, Alison M. Smith, Robert A. Field

**Affiliations:** From the ‡ John Innes Centre, Norwich Research Park, Norwich NR4 7UH, United Kingdom,; the § Institut für Biochemie und Biologie, Universität Potsdam, Karl-Liebknecht-Strasse 24-25, 14476 Potsdam-Golm, Germany,; the ¶ Department of Systems Biology, Technical University of Denmark, 2800 Lyngby, Denmark,; the ‖ Department of Plant Biology and Biotechnology, University of Copenhagen, Thorvaldsensvej 40, 1871 Frederiksberg C, Denmark, and; the ** Institute for Complex Systems and Mathematical Biology, University of Aberdeen, Meston Walk, Old Aberdeen, Aberdeen AB24 3UE, Scotland, United Kingdom

**Keywords:** Carbohydrate Metabolism, Computer Modeling, Metabolic Regulation, Oligosaccharide, Plant Biochemistry, Glucanotransferase, Leaf Cell, Maltose Metabolism, Starch Degradation

## Abstract

Controlled conversion of leaf starch to sucrose at night is essential for the normal growth of *Arabidopsis*. The conversion involves the cytosolic metabolism of maltose to hexose phosphates via an unusual, multidomain protein with 4-glucanotransferase activity, DPE2, believed to transfer glucosyl moieties to a complex heteroglycan prior to their conversion to hexose phosphate via a cytosolic phosphorylase. The significance of this complex pathway is unclear; conversion of maltose to hexose phosphate in bacteria proceeds via a more typical 4-glucanotransferase that does not require a heteroglycan acceptor. It has recently been suggested that DPE2 generates a heterogeneous series of terminal glucan chains on the heteroglycan that acts as a “glucosyl buffer” to ensure a constant rate of sucrose synthesis in the leaf at night. Alternatively, DPE2 and/or the heteroglycan may have specific properties important for their function in the plant. To distinguish between these ideas, we compared the properties of DPE2 with those of the *Escherichia coli* glucanotransferase MalQ. We found that MalQ cannot use the plant heteroglycan as an acceptor for glucosyl transfer. However, experimental and modeling approaches suggested that it can potentially generate a glucosyl buffer between maltose and hexose phosphate because, unlike DPE2, it can generate polydisperse malto-oligosaccharides from maltose. Consistent with this suggestion, MalQ is capable of restoring an essentially wild-type phenotype when expressed in mutant *Arabidopsis* plants lacking DPE2. In light of these findings, we discuss the possible evolutionary origins of the complex DPE2-heteroglycan pathway.

## Teaser

**Background:** Maltose metabolism during leaf starch degradation requires a multidomain glucanotransferase and a complex polysaccharide.

**Results:** A conventional bacterial glucanotransferase rescues an *Arabidopsis* mutant lacking the multidomain glucanotransferase.

**Conclusion:** Both the plant glucanotransferase-polysaccharide couple and the bacterial enzyme provide a glucosyl buffer in the starch degradation pathway.

**Significance:** New light is shed on the regulation and evolution of maltose metabolism.

## Introduction

Starch mobilization in leaves at night is one of the major metabolic fluxes in the biosphere. In many plants, up to half of the carbon assimilated via photosynthesis is stored as starch. The conversion of starch to sucrose during the night supplies the plant with carbon for metabolism and growth. In *Arabidopsis* grown in controlled conditions, night time conversion of starch to sucrose occurs at a constant rate that exhausts the starch reserves almost exactly at dawn. Mutants with reduced or accelerated rates of starch mobilization at night grow slowly and may exhibit symptoms of starvation. The main product of starch mobilization in the chloroplast is maltose, which is exported to the cytosol for conversion to hexose phosphates. These are used in cellular metabolism and for synthesis of sucrose for export to non-photosynthetic parts of the plant (1, 2). Despite the importance of night time starch mobilization for plant productivity, major questions remain over the pathway from maltose to hexose phosphate in the cytosol. The current state of knowledge is as follows.

**FIGURE 1.**
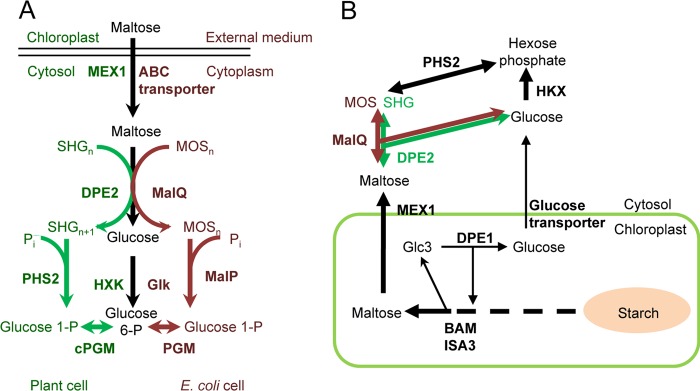
**Maltose metabolism in *E. coli* and *Arabidopsis* and the proposed pathway in the *Arabidopsis dpe2* mutant expressing MalQ.** *A*, in both *E. coli* and *Arabidopsis*, maltose enters the cytoplasm/cytosol, where it is disproportionated to yield glucose and a glucosylated acceptor molecule. Phosphorylation of glucose and phosphorolysis of the glucosylated acceptor then yield hexose phosphates. The pathway in *E. coli* is on the *right*; the putative pathway in leaf cells at night is on the *left. E. coli*-specific reactions are *dark red*, and plant-specific reactions are *green. B*, starch is metabolized to maltose and glucose in the chloroplast. Both metabolites are exported via specific transporters into the cytosol. In wild-type plants (*green*), maltose is disproportionated via DPE2 to yield glucose and glucosylated SHG. PHS2 can catalyze conversion of the glucosyl moiety to hexose phosphate. Free glucose is converted to hexose phosphate via hexokinase. In *dpe2*MalQ plants (*brown*), maltose is disproportionated via MalQ to yield glucose and malto-oligosaccharides. PHS2 can catalyze conversion of malto-oligosaccharides to hexose phosphate. As in wild-type plants, free glucose is converted to hexose phosphate via hexokinase. *MEX1*, maltose transporter. *MOS*, malto-oligosaccharide; *Glk*, glucose kinase; *MalP*, *E. coli* glucan phosphorylase; *BAM*, β-amylase; *ISA3*, isoamylase 3.

In the cytosol, maltose is acted on by a 4-α-glucanotransferase (DPE2), which is vital for the normal metabolism and growth of the plant. In *dpe2* mutants, maltose accumulates to very high levels, and growth is strongly reduced. DPE2 catalyzes the release of the reducing glucosyl moiety of maltose as free glucose, which can be phosphorylated via hexokinase to yield glucose 6-phosphate. The non-reducing glucosyl moiety of maltose is transferred onto an acceptor molecule (3–7). The nature of the acceptor *in vivo* is not known with certainty, but several lines of evidence indicate that it is a complex, soluble heteroglycan (SHG)^9^ widely distributed in plant species and organs (8–12). SHG is a heterogeneous mixture of glycans mainly composed of galactose and arabinose with minor amounts of glucose, fucose, mannose, rhamnose, and xylose. A defined proportion of SHG, which has been designated as subfraction I, comprises molecules larger than 10 kDa and is largely or exclusively cytosolic (8, 9, 13). *In vitro*, subfraction I both binds to recombinant DPE2 and acts as an acceptor of glucosyl moieties transferred by DPE2 from maltose. These glucosyl residues can be further metabolized by recombinant cytosolic phosphorylase isozyme (designated PHS2 in *Arabidopsis* and Pho2 in other species) to yield glucose 1-phosphate (6, 7). Thus, DPE2, cytosolic heteroglycan, and PHS2 together provide a route by which the non-reducing glucosyl moiety of maltose can be converted to hexose phosphate (Fig. 1*A*).

There is good circumstantial evidence that this pathway operates *in vivo*. First, mutant *Arabidopsis* and transgenic potato plants lacking DPE2 have up to 200 times more maltose than wild-type plants and reduced rates of starch degradation (3–5). In the Arabidopsis *phs2* mutant, maltose levels are elevated about 4-fold at night (7). Second, the structure of cytosolic heteroglycans is dependent on metabolic conditions. For example, the glucose content of these molecules in *Arabidopsis* leaves differs between night and day and is altered in *dpe2* mutants and lines with altered expression of cytosolic phosphoglucomutase (6, 10). Third, in short term experiments performed with potato tuber discs incubated with glucose 1-phosphate, the glucosyl content of cytosolic heteroglycans reflects the expression level of the cytosolic phosphorylase isozyme, Pho2 (10). Finally, there is no evidence for the existence in the cytosol of plant cells of glycogen or longer malto-oligosaccharides that could act as acceptors for the DPE2-mediated transfer of the non-reducing glucosyl residue from maltose. Soluble heteroglycans are the only cytosolic carbohydrates known to act as DPE2 acceptors.

The plant pathway of maltose metabolism is superficially similar to that of bacteria, including *Escherichia coli* (7, 14, 15). Maltose in *E. coli* is also metabolized to hexose phosphates via a 4-α-glucanotransferase (MalQ), a hexokinase, and a glucan phosphorylase (MalP) (Fig. 1*A*). The similarities are illustrated by the fact that an *E. coli* strain lacking MalQ was partially complemented with respect to growth on maltose by introduction of DPE2 from *Arabidopsis* (7). However, MalQ differs radically from DPE2 in that it appears to use maltose for a variety of transfer reactions leading to glucose and a series of malto-oligosaccharides (16, 17). There is no evidence that acceptors such as the cytosolic heteroglycans are involved in the bacterial pathway. Both enzymes belong to family 77 of glycoside hydrolases (GH77) (18), but whereas the GH77 domain is uninterrupted in MalQ, the domain in DPE2 contains an insertion of about 170 amino acids. DPE2 also possesses two N-terminal carbohydrate binding modules of the CMB20 family, but MalQ does not (19) (see Fig. 8*A*).

Little is known about the functional significance of the DPE2-cytosolic heteroglycan pathway in plants or the selective advantage that it confers over biochemically and genetically less complex pathways, such as that in *E. coli*. It has recently been proposed that cytosolic heteroglycan can act as a “glucosyl buffer” between maltose export from the chloroplast and hexose phosphate utilization in the cytosol (11). The combined action of a glucanotransferase and a phosphorylase both operating close to equilibrium is presumed to form a heterogeneous series of terminal glucan side chains of cytosolic heteroglycans. Modeling reveals that, due to heterogeneity in length (*i.e.* in glucosyl content), side chains permit temporary variation in one of these fluxes without significantly affecting the other flux (20). Such buffering may be vital for a steady and sustained supply of carbon from starch for night time metabolism in the leaf. However, there is no *a priori* reason why buffering between maltose and hexose phosphates should require proteins and substrates with the complexity of DPE2 and the cytosolic heteroglycan. It remains possible that these molecules have specific functions in the conversion of starch to hexose phosphate other than or in addition to providing a glucosyl buffer.

To shed light on the significance of the DPE2-cytosolic heteroglycan pathway, we compared key properties of DPE2 from *Arabidopsis* and MalQ from *E. coli*. We uncovered striking differences in substrate specificity and affinity, consistent with the idea that MalQ cannot use cytosolic heteroglycan as a substrate. However, simulations of our mathematical model demonstrated that a hypothetical plant pathway containing MalQ rather than DPE2 would retain a “glucosyl buffer” function without a requirement for cytosolic heteroglycan because, unlike DPE2, MalQ can convert maltose into a polydisperse series of malto-oligosaccharides. Thus, if the main function of the DPE2-cytosolic heteroglycan pathway *in vivo* is to provide a glucosyl buffer, introduction of MalQ might be expected to bypass the requirement for DPE2 *in planta*. To test this idea, we examined the impact of expression of MalQ in *dpe2* mutant plants. The transgenic plants regained metabolic functions and growth rates close to those of wild-type plants. The results have important implications for understanding the control of starch degradation and shed new light on the evolutionary origins of the starch degradation pathway.

## EXPERIMENTAL PROCEDURES

### 

#### 

##### Plant Growth and Transformation

Unless otherwise stated, *Arabidopsis thaliana* plants were grown in compost at 20 °C and 75% relative humidity with 12 h of dark, 12 h of light at 160 μmol quanta m^−2^ s^−1^. The *dpe2* mutant was *dpe2-3* (Wassilewskija, 3). For transformation, the MalQ gene from *E. coli* was synthesized with codon usage optimized for *Arabidopsis* (GeneArt®) and cloned into the vector plasmid pEarleyGate 202, which added a 35S promoter and an N-terminal FLAG tag to the protein and contained a glufosinate resistance gene cassette. The resulting plasmid was stably transferred into *dpe2-3* via *Agrobacterium tumefaciens* GV3101-mediated floral dip transformation. T0 plants were sprayed with glufosinate. Survivors were allowed to self-fertilize and were used to select homozygous lines.

##### Expression of Recombinant Proteins

Primers are shown in Table 1. *DPE2* (At2g40840) cDNA was as described (3). *PHS2* (At3g46970) cDNA was prepared from *Arabidopsis* (Col0) leaf mRNA with the primers described previously (7).

**TABLE 1:**
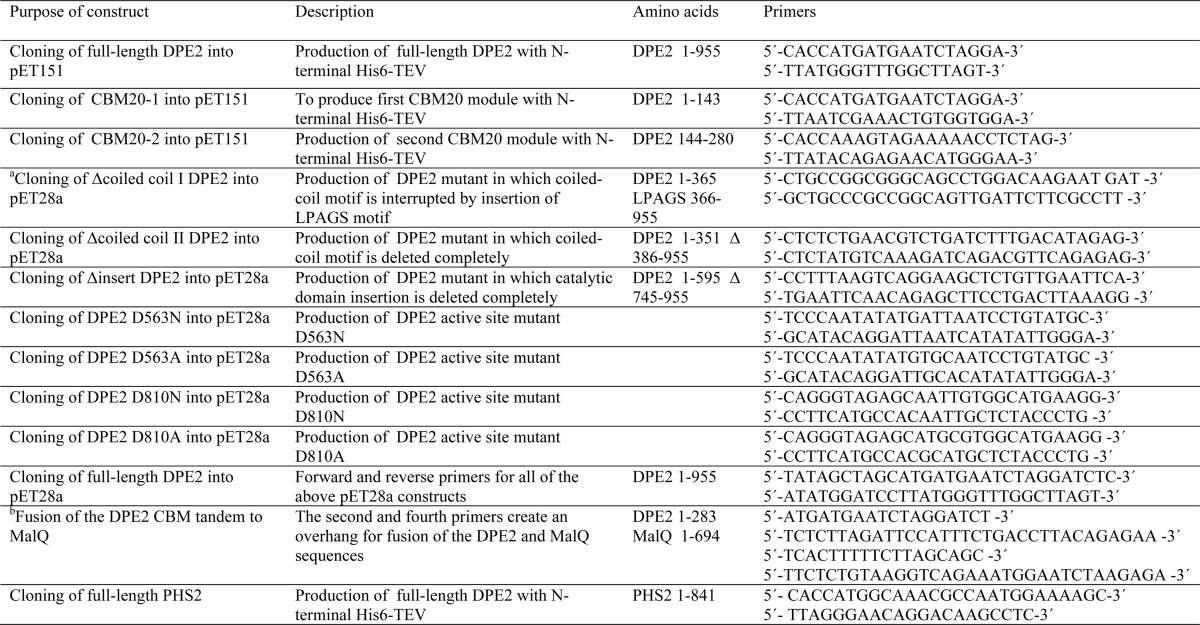
**Primers used in this study**

*^a^* For cloning into pET28a, the forward and reverse primers contain a NheI and a BamHI site, respectively. The primer pairs for pET28a constructs are mutation primers that either carry the respective mutation or contain sequences for insertions or deletions.

*^b^* MalQ in pMAD145 was a gift from Prof Winfried Boos (University of Konstanz).

For protein expression, cDNA was cloned into pET151 to give a construct encoding an isopropyl 1-thio-β-d-galactopyranoside-inducible, N-terminally His_6_-tagged protein with a TEV protease cleavage site C-terminal to the His tag. *E. coli* Rosetta II cells containing the constructs were grown overnight at 16 °C after induction. Cells were harvested by centrifugation and lysed with a cell disrupter in the presence of DNase, lysozyme, and Complete Protease Inhibitor EDTA-free (Roche Applied Science). After centrifugation and filtration to remove debris, extracts were subjected to chromatography on a HiTrap Chelating column (GE Healthcare), TEV protease treatment, and chromatography on a HiLoad Superdex S-200 16/60 preparation grade column (GE Healthcare), as described in the figure legends. Samples were loaded onto HiTrap columns in 100 mm HEPES (pH 7.5), 500 mm NaCl, 2 mm DTT, Complete Protease Inhibitor, and 60 mm imidazole, and bound proteins were eluted with this medium containing 500 mm imidazole. Superdex chromatography was in 25 mm HEPES (pH 7.5), 50 mm NaCl.

For dynamic light scattering, 20 μg of protein was filtered (0.1-μm membrane) and then analyzed in a 12-μl quartz cuvette in a dynamic light scattering device (DynaPro, Wyatt Technology Corp.). Measurements were taken every 5–10 s at 25 °C. Data were analyzed with the Dynamics^TM^ software package.

Circular dichroism measurements were made in a 0.1-mm quartz cuvette in a Jasco J-715 spectropolarimeter between 26 and 185 nm, in 0.5-nm steps. Data were analyzed with software from the DICHROWEB server.

##### Enzyme Assays

Unless otherwise stated, stopped assays of recombinant enzymes were in 100 μl of 100 mm Hepes (pH 7.0) at 37 °C, using enzyme and substrate concentrations stated in legends. Coupled assays in which glucose production was monitored were at pH 7.9, in the presence of 1 mm each of ATP, NAD, and MgCl_2_; 3 units of hexokinase; and 1 unit of glucose-6-phosphate dehydrogenase (NAD-linked, from *Leuconostoc mesenteroides*). Measurements from coupled assays were used to estimate *K_m_* values from Hanes plots.

##### Sugar Linkage Analysis

Analysis was as described previously (21, 22). Samples were per-*O*-methylated, hydrolyzed with trifluoroacetic acid, reduced with deuterated borohydride, and then per-*O*-acetylated with acetic anhydride. The resulting alditol acetates were solubilized in dichloromethane and analyzed by gas chromatography-mass spectrometry (23).

##### Binding of Recombinant Proteins to Starch

Starch granules were washed with 100 mm PIPES (pH 6.8) at 4 °C. Samples of 20 μm protein were mixed with 20 mg of starch/glucan in 500 μl of this buffer, shaken for 30 min on ice, and then centrifuged at 22,000 × *g* for 5 min. The pellet was washed extensively with phosphate-buffered saline and then incubated at 100 °C for 10 min in 500 μl of SDS-PAGE loading buffer. Soluble and pellet fractions were analyzed on 4–12% SDS-polyacrylamide gels.

##### Analysis of Actions on Maltose

Maltose (5 mm) in 50 μl of [^18^O]water was incubated for 5 min at 37 °C with 1 μg of recombinant protein or bovine serum albumin. The mixture was boiled and analyzed on a 100 × 2-mm Luna NH_2_ column (Phenomenex) run in HILIC (hydrophilic interaction liquid chromatography) mode at 200 μl min^−1^ and 30 °C with a gradient from 90 to 10% acetonitrile in 20 mm ammonium acetate (pH 9.45) over 15 min. Detection of [^18^O]glucose was by electrospray mass spectrometry.

##### Carbohydrate Microarrays

Polysaccharides (obtained either by enzymatic fragmentation of large cell wall polymers followed by purification or by chemical synthesis of various glycans) (24) were spotted onto nitrocellulose membranes (4 × 3 or 4 × 2 cm) using a microarray printer (24–26). Membranes were incubated with recombinant protein (5 mg) and ^14^C-labeled substrate (1 mm, either 37 GBq mol^−1^ (maltose) or 18 GBq mol^−1^ (glucose 1-phosphate)) in 5 ml of phosphate-buffered saline (PBS) for 4 h at 25 °C and then washed three times in PBS and twice in PBS with 0.05% (v/v) Tween 20 before fluorescence scanning in a PhosphorImager.

##### Surface Plasmon Resonance

Recombinant protein (2 mg ml^−1^) was mixed with a 20-fold molar ratio of 20 mm EZ-link Sulfo NHS-LC biotin (Pierce), incubated at room temperature for 30 min, and then passed twice through a Sephadex G20 gel filtration spin column (GE Healthcare). Eluted protein at 10 μg ml^−1^ in 10 mm HEPES (pH 7.0), 150 mm NaCl was applied to a Series S sensor chip SA (GE Healthcare), activated with 50 mm NaOH, 1 m NaCl, to give 3000 bound response units. For measurements with maltotriose and maltotetraose, recombinant MalQ was directly immobilized on a Series S Sensor chip CM5 activated with 1-ethyl-3-(3-dimethylaminoproplyl)-carbodiimide and *N*-hydroxysuccinimide. Protein was applied at 100 μg ml^−1^ in 10 mm sodium acetate (pH 5.0), 150 mm NaCl and immobilized with an amine coupling kit (GE Healthcare) to give 6000 bound response units. The surface was then quenched with ethanolamine.

Analytes were applied to chips at 30 μl min^−1^ at 25 °C in 10 mm HEPES (pH 7.0). Contact time was 90 s; dissociation time was 30 s; and regeneration was with 10 mm HEPES (pH 7.0), 1 m NaCl for 90 s. Measurements were in triplicate on each of two chips. Analyte concentrations ranged from 50 nm to 10 mm. Experiments were performed on a Biacore T100 (GE Healthcare).

##### Experiments with SHG_L_

SHG_L_ was purified from *Arabidopsis* (Ws) leaves harvested at the end of the light period as described previously (13). For experiments with unlabeled SHG_L_ and labeled maltose, samples of 30 μg were incubated in 150 μl of 100 mm citrate/NaOH (pH 7.0), 5 mm MgCl_2,_ 10 mm dithioerythritol with recombinant protein and 12 mm [^14^C]maltose at 37 °C. For experiments with [^14^C]SHG_L_, prelabeling was by incubation of 40 μg of SHG_L_ with 6 μg of recombinant DPE2 and 12 mm [^14^C]maltose in 100 μl at 37 °C, followed by heating (95 °C for 5 min), centrifugation (20,000 × *g* for 10 min at 4 °C), and washing on a 10-kDa membrane filter. Samples of 20 μg of [^14^C]SHG_L_ were incubated with recombinant protein as above except that maltose was 10 mm and unlabeled. After incubations, mixtures were centrifuged and washed on a filter as above. Radioactivity in the filtrate and retentate was measured. For experiments in which neither SHG_L_ nor maltose was labeled, incubations contained recombinant MalQ, 200 μg of SHG_L_ (∼8 nmol of glucosyl moieties), and 8 nmol of maltose. After incubation, the mixture was heated, centrifuged, and passed through a 10-kDa filter. The filtrate was analyzed by HPAEC-PAD.

##### Native Gels for Enzyme Activity

For DPE2 and MalQ activity, leaves (∼1 g) were harvested into liquid N_2_ midway through the light period (unless stated otherwise), powdered in liquid N_2_, and then homogenized in 5 ml of 100 mm MOPS (pH 7.0), 150 mm NaCl, 10% (v/v) glycerol, 0.1% (v/v) Triton X-100, 50 μm DTT, and 10 μl ml^−1^ protease inhibitor (Roche Applied Science) at 4 °C and centrifuged at 20,000 × *g*. For PHS2 activity, extraction was as above except the medium was 2 ml of 100 mm Tricine-NaOH (pH 7.8), 5 mm MgCl_2_, 10 mm dithioerythritol, 0.5 mm PMSF, 2 mm benzamidine, 2 mm aminocaproic acid, 10% (v/v) glycerol. Samples (2.5 μg of protein for DPE2 and MalQ, 10 μg for PHS2) were mixed with native sample buffer and loaded onto acrylamide gels containing 0.5% (w/v) glycogen (for DPE2), 0.2% (w/v) glycogen (for PHS2), or no glycogen (for MalQ). After electrophoresis, gels were washed with 100 mm MOPS (pH 7.0) (for DPE2 and MalQ) or 100 mm citrate-NaOH (pH 6.5) (for PHS2) and then typically incubated for 2 h at 37 °C in the same buffer plus 5 mm maltose for DPE2, 5 mm Glc7 for MalQ, or 20 mm glucose 1-phosphate for PHS2, prior to staining with aqueous 0.67% (w/v) I_2_ and 3.33% (w/v) KI.

##### Carbohydrate Analyses

A representative 50–150-mg sample of a single rosette was harvested into liquid N_2_ and powdered in a ball mill. Extraction in dilute perchloric acid, starch solubilization and hydrolysis, enzymatic assay of sucrose and hexoses, and analysis of malto-oligosaccharides by HPAEC-PAD were as described previously (27, 28).

##### Numeric Simulations

The numeric algorithms simulate the reaction systems by a discrete number of oligoglucan molecules and enzymes that interact through enzyme-substrate complex formation and enzymatically catalyzed chemical conversions. In analogy to classical thermodynamics, each glucan G*_n_*, *n* denoting the degree of polymerization, can be interpreted as a defined energy state that may be occupied by an arbitrary number of particles (see Ref. 20). The stochastic algorithms used to simulate all reaction systems proceed according to the scheme introduced by Gillespie (29). The propensities determining the probabilities of which event is executed in the next simulation step and the time until this event occurs are determined by the rate constants through mass action rate laws.

The enzyme MalQ catalyzes the general reaction scheme, G*_n_* + G*_m_* → G_*n* − *q*_ + G_*m* + *q*_, where it is assumed that maltose (*n* = 2) cannot act as a glucosyl donor. Various experimental findings indicate that MalQ endogenously binds glucosyl units that may be used as donors to elongate malto-oligosaccharides, thus explaining the observed time lag when MalQ is incubated with pure maltose. This hypothesis was tested *in silico* by simulating the action of MalQ with a corresponding reaction mechanism. A MalQ enzyme with *k* glucosyl residues intrinsically bound is denoted *E_k_*. The simulation of the MalQ reaction mechanism implements the following reaction steps: 1) transfer of glucosyl residues from glucan to enzyme,

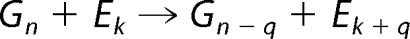
 and 2) transfer of glucosyl residues from enzyme to glucan,

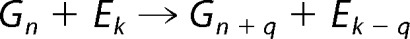
 It is assumed that maltose cannot act as a donor; therefore, the reaction, *G*_2_ + *E_k_* → *G*_1_ + *E_k_*_+ 1_, is forbidden. Due to thermodynamic reasons, the reverse reaction, *G*_1_ + *E_k_* → *G*_1_ + E_*k* − 1_, must also be forbidden. Except for these forbidden reaction steps, it is assumed that all possible transfer reactions proceed according to a bilinear rate law with identical second order rate constants, *k*_MalQ_.

##### Simulations of the NAD(H)-coupled MalQ Assays

For the simulation of the NAD(H)-coupled *in vitro* assays of MalQ, the hexokinase reaction,

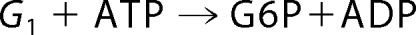
 was additionally represented as a simple first-order reaction with rate constant *k*_HXK_, corresponding to the assumption that ATP concentration is large and remains approximately constant throughout the reaction and that the reaction is practically irreversible under the chosen conditions. The number of glucose 6-phosphate molecules produced was recorded and plotted in Fig. 9*A*. The following initial conditions and parameters were used for the *in vitro* simulation: at time *t* = 0, *G*_2_ = 10,000, *G_n_* = 0 for n ≠ 2, *E*_1_ = 59, *E*_2_ = *E*_3_ = 2, *E*_4_ = *E*_5_ = *E*_6_ = 1; rate constants, *k*_MalQ_ = 0.0006 s^−1^, *k*_HXK_ = 0.02 s^−1^.

##### Simulations of the MalQ/PHS2 Buffer

To simulate an *in vivo* function of the MalQ-mediated buffer, the phosphorylase PHS2 was also represented in the simulations. The forward and backward reactions

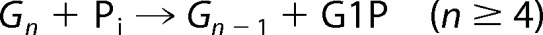


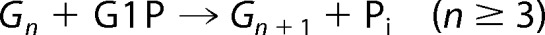
 were represented as second order mass action reactions. The ratio of the forward and backward rate constants *k*^+^_PHS_ and *k*^−^_PHS_ was assumed to be *K*_eq_ = *k*^+^_PHS_/*k*^−^_PHS_ = 0.19, in agreement with the findings reported in Ref. 20. The concentration of inorganic phosphate was assumed to be constant. Further, for the sake of simplicity, no distinction was made between glucose 1-phosphate and glucose 6-phosphate, and both were lumped into one variable representing the total pool of glucose phosphates.

To simulate an open system, an influx of maltose from an external source was represented as a zero order reaction (rate *v*_in_), and a consumption of glucose phosphate was represented as a first order reaction with rate constant *k*_out_.

For Fig. 9, *B–D*, maltose influx was simulated as a fluctuating function according to the equation,

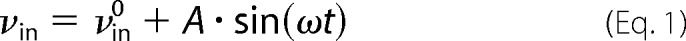
 The following parameter values were used: *k*_MalQ_ = 0.01 s^−1^; *k*_HXK_ = 0.1 s^−1^; *k*^+^_PHS_ = 10^−5^ s^−1^; *k*_out_ = 0.2 s^−1^; *v*_in_ = 100 s^−1^; *A* = 100 s^−1^; ω = 0.2 s^−1^ (rapid), 0.02 s^−1^ (medium), or 0.002 s^−1^ (slow fluctuations); P_i_ = 50,000.

## RESULTS AND DISCUSSION

### 

#### 

##### Diverse Heteroglycans Are Acceptors for DPE2 but Not MalQ

In *in vitro* studies, recombinant DPE2 uses glycogen and cytosolic heteroglycans as acceptors for the transfer of the non-reducing glucosyl moiety from maltose. In addition, a glucosyl transfer from glycogen to monosaccharides, including mannose and xylose, was demonstrated (3, 6). These kinetic properties are consistent with the idea that DPE2 transfers glucosyl moieties from maltose onto glucosyl and other terminal residues of cytosolic heteroglycan *in vivo*. We investigated whether MalQ shares this broad acceptor specificity.

**FIGURE 2.**
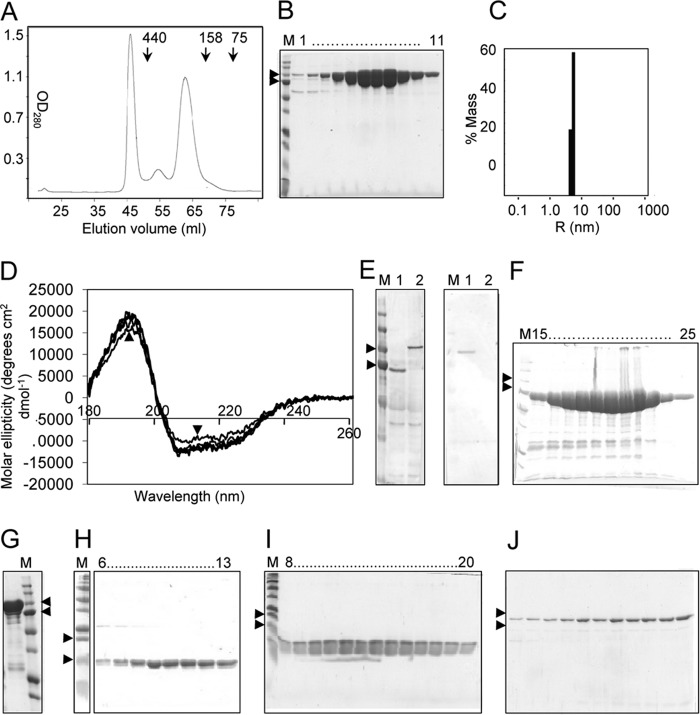
**Purification of recombinant proteins from extracts of *E. coli* cells.** *A–C*, DPE2 purification. *A*, protein elution profile from a Superdex S200 column following nickel immobilized metal affinity chromatography (IMAC), His tag cleavage with TEV protease, and then a second IMAC step; unbound protein from this step was loaded onto the column. Activity coincided with the peak of protein at 58–68 ml. Elution positions of molecular mass markers (kDa) are indicated. *B*, SDS-polyacrylamide gel (12.5%) of fractions 58–68 from *A. C*, dynamic light scattering analysis of purified protein. The protein (10 mg ml^−1^) was monitored for 10 s at 25% laser intensity (∼3,000,000 counts s^−1^). Values are means from 10 readings. *D*, analysis of native and mutant forms of DPE2 by circular dichroism polarimetry. Measurements from 180 to 260 nm were used to calculate molar ellipticity. *Lines* represent the native protein (*arrow*) and the D563N, D563A, D810N, and D810A mutant forms. *E*, polyclonal antibodies were raised in rabbits by Eurogentec (Southampton, UK) by immunization with purified DPE2 or MalQ. Antisera were purified on affinity columns of pure DPE2 or MalQ coupled to aldehyde-activated beaded agarose, followed by elution at pH 2.5. Eluted fractions were tested on blots of SDS-polyacrylamide gels of transgenic plants expressing either MalQ or CBM-MalQ (*left*, *lanes 1* and *2*) for the MalQ antiserum (1:3000 dilution) or extracts of wild-type and *dpe2* mutant plants (*right*, *lanes 1* and *2*) for the DPE2 antiserum (1:5000 dilution). *F*, MalQ purification. SDS-polyacrylamide gel (12.5%) of His-tagged protein in fractions from IMAC. *G*, CBM20-MalQ purification; like *F* but showing unbound protein from IMAC purification after an initial IMAC step followed by His tag cleavage with TEV protease. *H*, CMB20-1 purification; SDS-polyacrylamide gel (15%) of fractions from a Superdex S-200 column loaded with peak fractions from an initial IMAC step. *I*, CBM20-2 purification; like *H*, but the Superdex S-200 step was preceded by an initial IMAC step, His tag cleavage with TEV protease, and then a second IMAC step. *J*, PHS2 purification; SDS-polyacrylamide gel (12.5%) of fractions from a Sephacryl S-200 column of His-tagged protein purified by single step IMAC as described above. *M*, molecular mass markers. *Arrows*, 100 and 75 kDa (*B*, *E*, *F*, *G*, and *J*) or 20 and 15 kDa (*H* and *I*).

**FIGURE 3.**
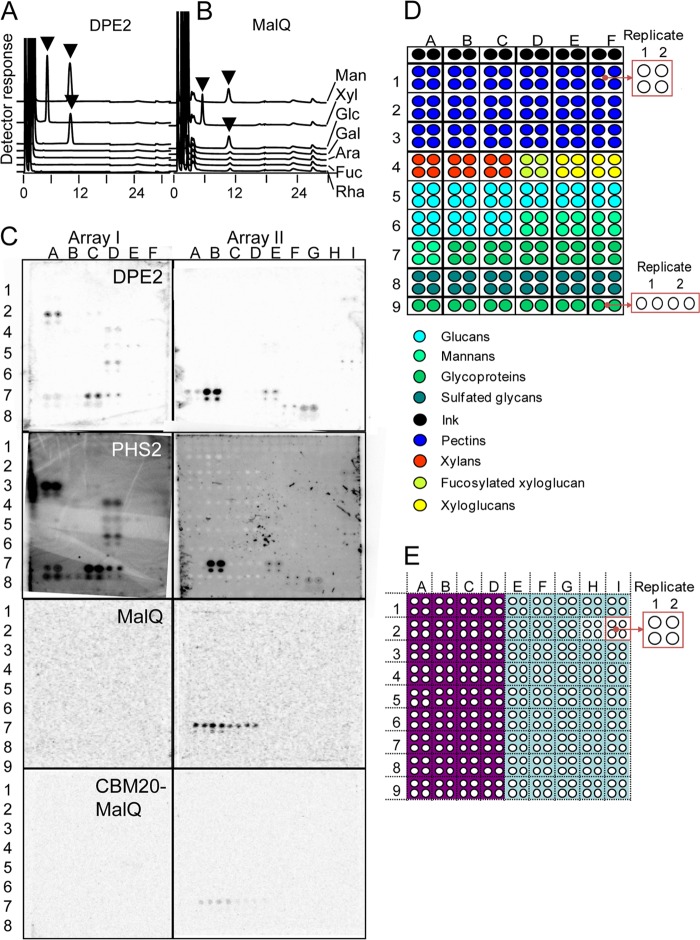
**Acceptor substrates for glucosyl transfer by DPE2 and MalQ.** *A* and *B*, HPAEC profiles of the products of glucosyl transfer from glycogen to monosaccharides. Recombinant DPE2 (*A*) and MalQ (*B*) (2.5 μg each) were incubated for 90 min at 37 °C with glycogen and various monosaccharides (30 mm). Reaction mixtures were then passed through a 10-kDa cut-off filter, and the filtrates were analyzed by HPAEC-PAD. The *x* axis shows elution time (min). *Arrows*, disaccharides. The same method also revealed formation of disaccharides by both enzymes with *N*-acetyl-d-glucosamine, allose, and 2,3-dideoxy-d-glucose. For both enzymes, disaccharides formed from glucose and xylose were shown to be α1,4-linked by MS fragmentation of partially methylated alditol acetates derived from the disaccharides (not shown). *C*, glucosylation of arrayed carbohydrates by recombinant enzymes. Malto-oligosaccharides and fragments of plant cell wall polysaccharides were printed onto nitrocellulose membranes (array I, 4 × 2 cm; array II, 4 × 3 cm). Membranes were incubated with recombinant proteins (as indicated on each pair of arrays) in 5 ml of buffer. Concentrations of proteins and ^14^C substrates were as follows: 1 mg ml^−1^ DPE2 and [^14^C]maltose; 1 mg ml^−1^ PHS2 and [^14^C]glucose 1-phosphate; 1 mg ml^−1^ MalQ and [^14^C]maltose; 1 mg ml^−1^ CBM20-MalQ and [^14^C]maltose. After a 4-h incubation at 25 °C, the membranes were washed, dried, and imaged on a PhosphorImager. *D*, layout of array I, shown at the *left* in *C*. Classes of compounds are *color-coded* as shown. The nature of the compounds is given in Table 2. For each replicate, glycan was applied at 0.5 mg ml^−1^ (*top two spots* or *left two spots* for the *bottom row*) and 0.1 mg ml^−1^ (*bottom two spots* or *right two spots* for the *bottom row*). *E*, layout of array II, shown at *right* in *C*. The nature of the compounds is given in Table 3. *Purple*, compounds applied as conjugates with bovine serum albumin. For each replicate, glycan was applied at 0.5 mg ml^−1^ (*top two spots*) and 0.1 mg ml^−1^ (*bottom two spots*).

Recombinant DPE2 and MalQ (purification shown in Fig. 2, *A*, *B*, and *F*) both formed disaccharides with d-glucose, d-mannose, d-xylose, d-allose, *N*-acetyl-d-glucosamine, and 2,3-dideoxy-d-glucose but not with l-rhamnose, d-galactose, d-fucose, or l-arabinose (Fig. 3, *A* and *B*). Thus, both DPE2 and MalQ can use a relatively wide range of monosaccharides as glucosyl acceptors. Consideration of the structures of these sugars suggests that efficient monosaccharide acceptors have both a d-configured pyranose ring and an equatorial orientation of the hydroxyl group at C4.

To investigate the actions of DPE2 and MalQ on complex polysaccharides, we used carbohydrate microarrays consisting of fragments of plant and algal carbohydrates printed onto nitrocellulose membranes (24–26, 30) (Tables 2 and 3). After incubation with recombinant protein and [^14^C]maltose, membranes were washed to minimize nonspecific binding and then subjected to fluorescence scanning. Recombinant DPE2 transferred ^14^C from [^14^C]maltose onto diverse carbohydrates, including the sulfated algal carbohydrate carrageenan, rhamnogalacturonan I, rhamnogalacturonan II-enriched pectin, fucosylated xyloglucan, cellulose, glucomannan, and β 1,3-linked d-glucan (Fig. 3*C*). In contrast, recombinant MalQ transferred ^14^C from [^14^C]maltose only onto α1,4- and α1,4-, α1,6-linked glucans (maltose, maltopentaose, α-(1,6)-d-glucosyl-α-(1,4)-d-maltotriose, and α-(1,6)-d-glucosyl-α-(1,4)-d-maltosyl-maltose) (Fig. 3*C*). Neither of the latter two branched oligoglucans was used by DPE2 as an acceptor.

**TABLE 2:**
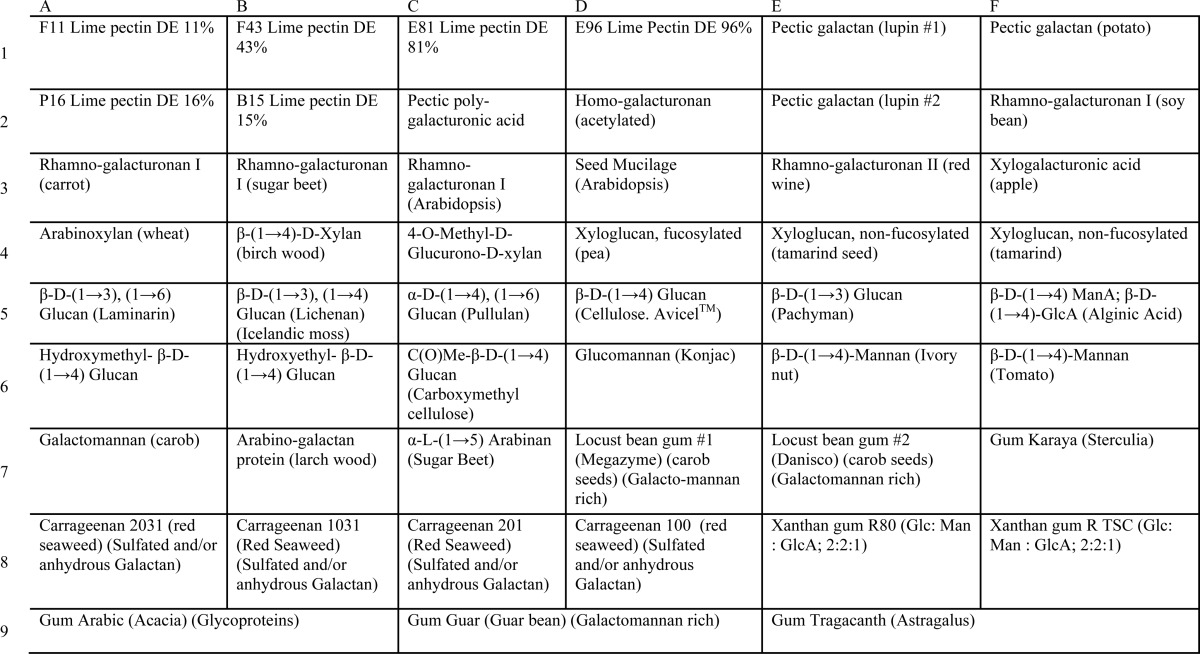
**Layout of carbohydrate microarray I** Glycans on the array shown at the *left* in Fig. 3*C* and in Fig. 3*D* are listed.

**TABLE 3:**
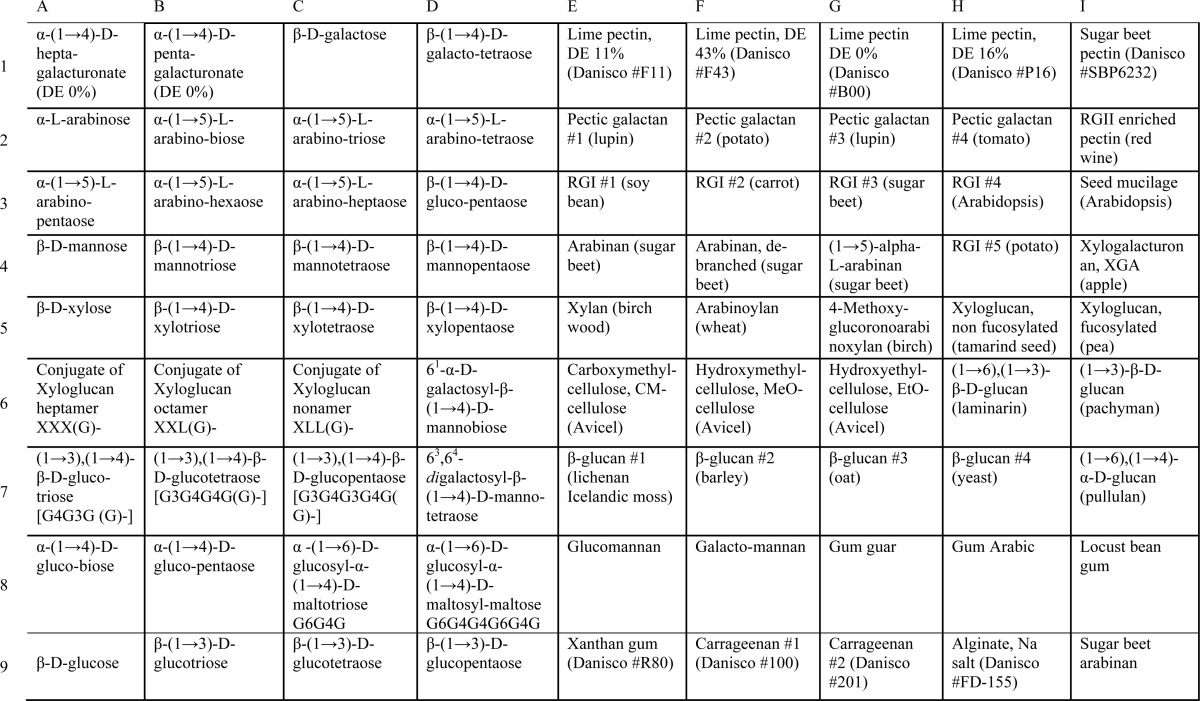
**Layout of carbohydrate microarray II** Glycans on the array shown at the *right* in Fig. 3*C* and in Fig. 3*E* are listed.

We also used the carbohydrate microarrays to examine the acceptor specificity of the cytosolic phosphorylase isozyme PHS2. Recombinant PHS2 mediates the reversible glucosyl transfer between cytosolic heteroglycans and glucose 1-phosphate *in vitro* (6–9, 13). The enzyme (purification shown in Fig. 2*J*) transferred ^14^C from [^14^C]glucose 1-phosphate to diverse carbohydrate fragments on the microarrays. Intriguingly, the pattern of labeling was very similar to that of DPE2, including labeling of carrageenan, rhamnogalacturonan I, rhamnogalacturonan II, and fucosylated xyloglucan (Fig. 3*C*). These data are consistent with the idea that DPE2 and PHS2 may act on a common, complex polysaccharide substrate *in vivo*.

**FIGURE 4.**
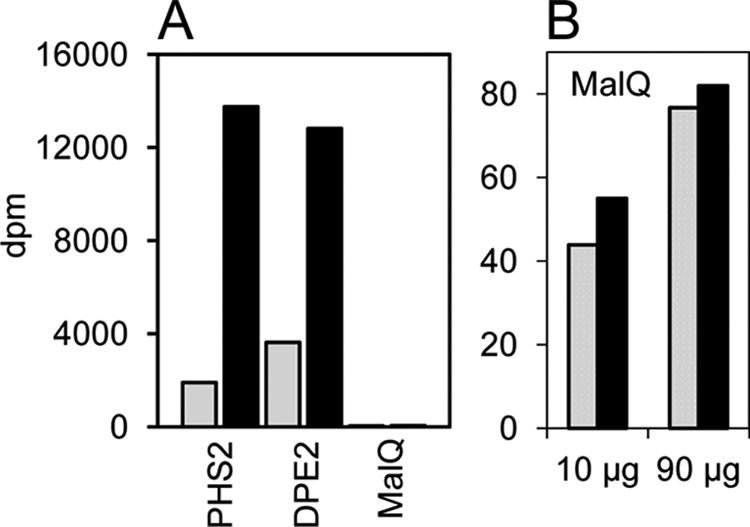
**Transfer of glucosyl residues onto SHG_L_ by PHS2, DPE2, and MalQ.** Purified SHG_L_ was incubated with recombinant enzyme and either [^14^C]maltose (for DPE2 and MalQ) or [^14^C]glucose 1-phosphate (for PHS2) for 10 min (*gray bars*) or 60 min (*black bars*), and then dpm in SHG_L_ were determined. Values are means of values from two separate incubations. *A*, incorporation catalyzed by 10 μg of each enzyme. *B*, incorporation catalyzed by 10 and 90 μg of MalQ. Values for 10 μg are the same as those shown in *A*. Note the very low values and lack of proportionality of incorporation with time or amount of enzyme.

**FIGURE 5.**
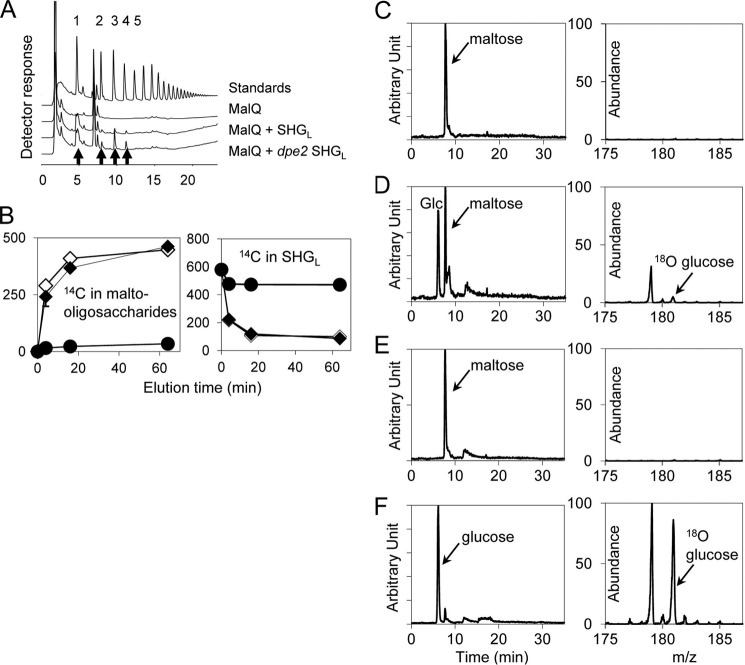
**MalQ-mediated reactions.** *A* and *B*, release of malto-oligosaccharides from SHG_L_ by recombinant MalQ. *A*, MalQ was incubated for 90 min alone or with SHG_L_ purified from either wild-type or *dpe2* mutant plants. After the removal of protein and SHG_L_, products were subjected to HPAEC. Glucose (*left*) and malto-oligosaccharide products formed in the presence of SHG_L_ are indicated with *arrows*. Note that SHG_L_ from *dpe2* mutant plants contains more glucose (*Glc*) than that from wild-type plants (6). *B*, SHG_L_ purified from wild-type plants was labeled by incubation for 10 min with DPE2 and [^14^C]maltose. After the removal of protein and maltose, the [^14^C]glucosyl-SHG_L_ was incubated with MalQ for 4, 16, or 64 min, with or without unlabeled maltose. ^14^C released as malto-oligosaccharides (*left*) and remaining in SHG_L_ (*right*) was measured. *Filled diamonds*, without maltose. *Open diamonds*, with maltose. *Circles*, control with maltose and no MalQ. Data are typical of those obtained in several independent experiments. Results were similar when SHG_L_ was labeled for 40 min prior to incubation with MalQ. *C–F*, analysis of the actions of DPE2 and MalQ on maltose. Maltose (5 mm) was incubated for 5 min at 37 °C with protein (0.02 μg μl^−1^) and ^18^O water. Products were analyzed by hydrophilic interaction chromatography (elution profiles on *left*; *y* axis values based on peak area of the maltose substrate) and electrospray mass spectrometry (*right graphs*; data are expressed relative to the *unlabeled peak* in *F*). *C*, DPE2. *D*, MalQ. *E*, bovine serum albumin. *F*, yeast α-glucosidase. *Glc*, glucose.

The fact that MalQ used only α1,4- and α1,4-, α1,6-linked d-glucans as substrates on the carbohydrate microarrays indicated that, unlike DPE2, it may be unable to use plant cytosolic heteroglycans as a substrate. To test this idea, SHG_L_ isolated from wild-type *Arabidopsis* leaves was incubated with recombinant MalQ and [^14^C]maltose. Incorporation of ^14^C into SHG_L_ was extremely low (Fig. 4). In contrast, and as expected (6, 13), both DPE2 and PHS2 labeled SHG_L_ extensively (from [^14^C]maltose and [^14^C]glucose 1-phosphate, respectively). After 60 min of incubation with 10 μg of recombinant protein, labeling of SHG_L_ via DPE2 and PHS2 was 230 and 560 times greater, respectively, than labeling via MalQ. In further experiments, we examined whether MalQ has significant hydrolytic activity toward either SHG_L_ or maltose. We found that the enzyme can release glucose and malto-oligosaccharides from preglucosylated SHG_L_ in the absence of any acceptor other than water (Fig. 5, *A* and *B*). This action may be hydrolytic, but as we discuss below, it might also reflect the possession by MalQ of tightly bound malto-oligosaccharides that can act as initial substrates of the reaction. MalQ has very limited hydrolytic activity on maltose, as demonstrated by ^18^O labeling of glucose by recombinant MalQ in the presence of maltose and [^18^O]water (Fig. 5, *C–F*), but by far its predominant action on maltose is disproportionation (see below).

These results show that although MalQ and DPE2 have similar, relaxed specificities toward monosaccharides as acceptors, only DPE2 can use complex heteroglycans as acceptors. MalQ exclusively uses α-glucans as acceptors.

##### Maltose and Small Malto-oligosaccharides Are Good Acceptors for MalQ but Not DPE2

DPE2 and MalQ differ profoundly in their actions on small malto-oligosaccharides. It has been reported that DPE2 has no activity on maltose alone, whereas MalQ can use maltose as both donor and acceptor. Thus, MalQ acts on maltose when added as the only exogenous carbohydrate, leading to the formation of glucose and a series of malto-oligosaccharides. By contrast, DPE2 efficiently metabolizes maltose only when provided with a distinct acceptor molecule (6, 16, 17, 19). To probe these differences further, we compared the actions of MalQ and DPE2 on malto-oligosaccharides of different lengths.

**FIGURE 6.**
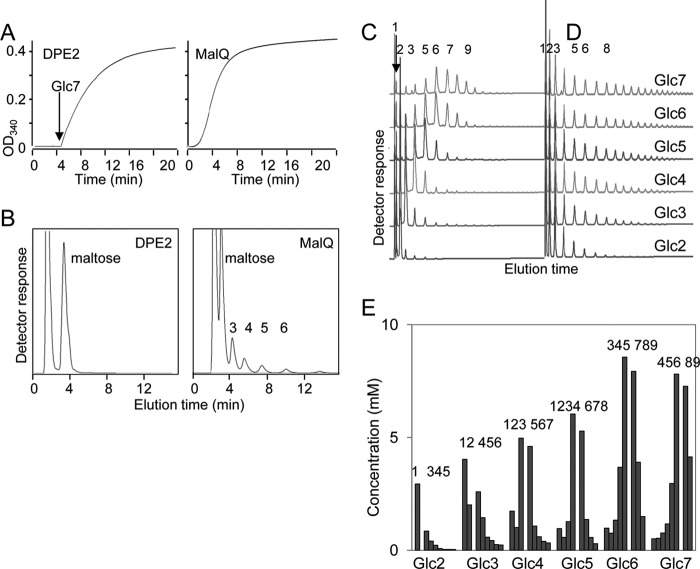
**Disproportionation of linear malto-oligosaccharides by DPE2 and MalQ.** *A* and *B*, short incubations of recombinant enzymes (0.25 μm DPE2, 0.33 μm MalQ) with 0.05 mm maltose. *A*, release of glucose. Note release after a short lag in the presence of MalQ but not DPE2. For the DPE2 incubation, Glc7 (2.5 mm) was added after 5 min to confirm that the enzyme was active. *B*, products of 10-min incubations, analyzed by HPAEC. *C* and *D*, recombinant enzymes (1 μm each) were incubated with malto-oligosaccharides (maltose to Glc7; 25 mm) for 12 h at 37 °C. Products were analyzed by HPAEC. The *numbers above* the *peaks* show the degree of polymerization of the products. *C*, DPE2; *D*, MalQ; *E,* quantification of data shown in *C.* Products were quantified by reference to pure malto-oligosaccharide standards. *Numbers above bars* show the degree of polymerization of the major products for each substrate.

In short incubations, no disproportionation of maltose was detected with DPE2 (Fig. 6, *A* and *B*). In long incubations at high enzyme concentrations, both enzymes disproportionated all of these molecules, although DPE2 action on maltose was very limited (Fig. 6, *C* and *D*). From each malto-oligosaccharide, MalQ produced glucose and a series of longer malto-oligosaccharides, suggesting that the initial products of disproportionation were themselves further disproportionated and that the reaction reached a thermodynamic equilibrium (20). However, DPE2 produced predominantly malto-oligosaccharides differing in size from the starting molecule by one glucosyl residue (Fig. 6, *C* and *E*). Thus, for example, DPE2 produced predominantly maltopentaose (Glc5) and maltoheptaose (Glc7) when incubated with maltohexaose (Glc6). Incubations with maltotriose (Glc3) were an exception; the main products were glucose and maltotetraose (Glc4) rather than maltose and Glc4. This distribution probably reflects the use of the maltose product as a donor for glucosylation of Glc3 to produce glucose and Glc4. Indeed, maltose was one of the least abundant DPE2 products in all incubations. This experiment shows that DPE2 preferentially transfers single glucosyl residues, consistent with the restriction of substrate binding beyond subsite −1, as proposed previously (19).

**FIGURE 7.**
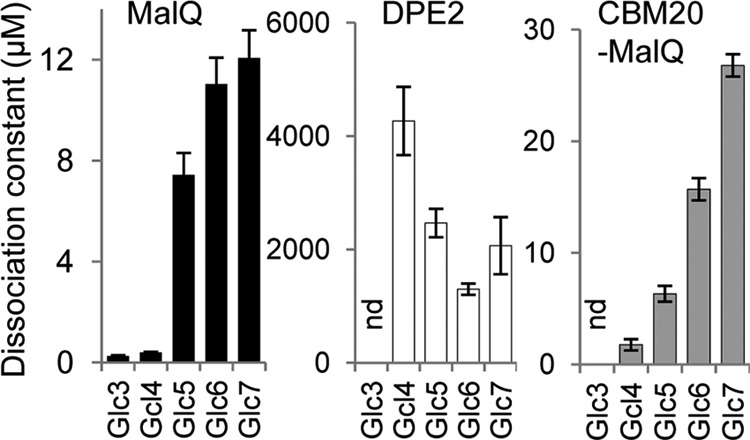
**Affinity for malto-oligosaccharides of DPE2, MalQ, and a CBM20-MalQ fusion protein.** SPR sensorgrams of the binding of 50 nm to 10 mm malto-oligosaccharides (Glc3 to Glc7) were recorded. For most measurements, recombinant proteins were biotinylated for coupling to a streptavidin SPR chip. For measurements of binding of MalQ to Glc3 and Glc4, the protein was coupled via its amino groups to a CM5 chip; this allowed higher protein densities to be used, producing a greater signal. Dissociation constants were calculated by steady-state affinity fitting (BIAevaluation version 2.0.3 software) to the response after subtracting the reference cell signal and a buffer blank. Note the different *y* axis *scales. nd*, not determined. *Error bars*, S.D.

We used surface plasmon resonance (SPR) to measure binding affinities of MalQ and DPE2 for malto-oligosaccharides. Immobilized recombinant proteins were exposed to various concentrations of malto-oligosaccharides. MalQ displayed a very high affinity (*i.e.* low dissociation constant) for Glc3 and Glc4 and progressively lower affinities for Glc5, Glc6, and Glc7. DPE2 had much lower affinity for malto-oligosaccharides in general and bound malto-oligosaccharides Glc5, Glc6, and Glc7 more strongly than Glc4 (Fig. 7). The dissociation constant of MalQ for Glc4 was more than 10,000 times lower than that of DPE2, whereas its dissociation constant for Glc7 was only 170 times lower than that of DPE2. Overall, these data show that MalQ can rapidly generate a wide range of malto-oligosaccharides from single, small malto-oligosaccharide species, including maltose, whereas DPE2 has only a very low affinity for and action on small malto-oligosaccharide acceptors.

Despite these profound differences in acceptor specificity, we found that MalQ and DPE have very similar affinities for maltose as a donor. For two separate preparations of the enzymes, MalQ had *K_m_* values of 2.1 and 1.9 mm (with Glc7 as an acceptor), and DPE2 had *K_m_* values of 2.9 and 2.3 mm (with glycogen as an acceptor).

##### The CBM20 Domain Confers Some but Not All of the Differences in Substrate Specificity

**FIGURE 8.**
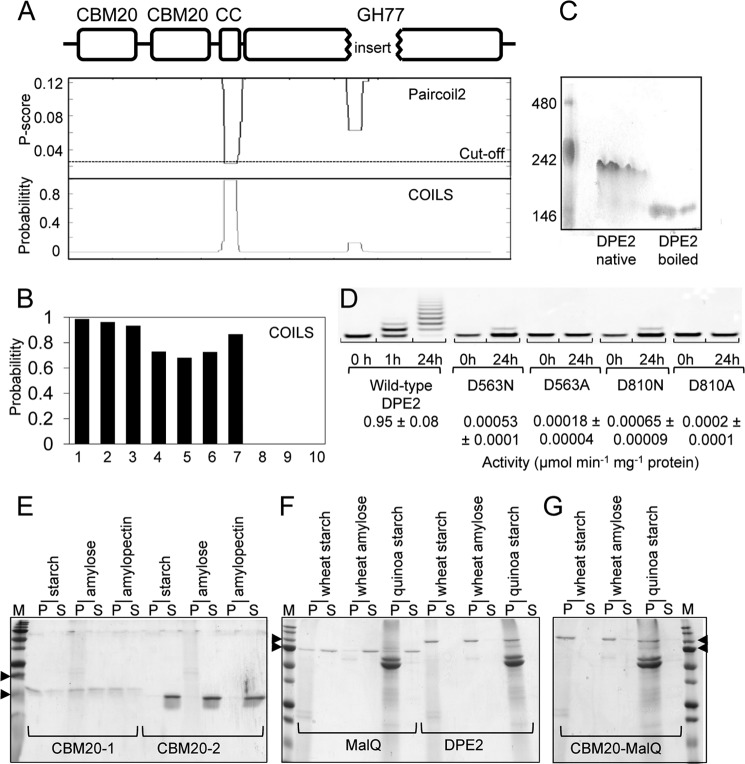
**Structure-function relationships of DPE2.** *A*, amino acid sequence of DPE2 analyzed for coiled-coils with programs Paircoil2 and COILS (window frame 28). High likelihood of a coiled-coil is implied by a low *p* score in Paircoil2 and a high probability score in COILS. The predicted domain structure of DPE2 is shown *above. CC*, predicted coiled-coil motif. *B*, COILS analysis of DPE2 sequences from *A. thaliana*, *Hordeum vulgare*, *Populus trichocarpa*, *Annona cherimola*, *Solanum tuberosum*, *Selaginella moellendorfii*, and *Ostreococcus lucimarinus* (*bars 1–7*, respectively); DPE1 sequence from *A. thaliana* (*bar 8*); and MalQ sequence from *E. coli* and *Thermotoga maritima* (*bars 9* and *10*). *C*, native PAGE (7.5%) of native or boiled DPE2 (2.5 μg). Markers are ferritin (480 kDa), β-phycoerythrin (242 kDa), and lactate dehydrogenase (146 kDa). *D*, effects of mutations in the catalytic domain; fluorophore-assisted carbohydrate electrophoresis of products of DPE2 (1.5 μg) with 1 mm APTS-derivatized Glc7 and 5 mm maltose. Separation was on 30% polyacrylamide gels in Tris borate at 450 V, and visualization was by scanning at emission and excitation wavelengths of 502 and 426 nm, respectively. Samples were wild-type enzyme or mutant forms with Asp-563 or Asp-810 changed to either alanine or asparagine, as indicated. *Numbers below* are the initial rates of glucose production from 2 mm maltose and 10 mm Glc7 (means ± S.D. (*error bars*); *n* = 3). *E–G*, affinity for starch and starch polymers. Starch, amylose, or amylopectin (20 mg; potato unless otherwise indicated) was incubated with recombinant proteins (20 μm) for 30 min at 4 °C. Pellet (*P*) and supernatant (*S*) fractions were separated by centrifugation and analyzed by SDS-PAGE (4–12% gradient gels). Extra bands in quinoa starch are starch-intrinsic proteins. *Arrows* in *E*, 20 and 15 kDa markers; *arrows* in *F* and *G*, 75 and 100 kDa markers.

We investigated which structural features of the enzymes may be responsible for the very different acceptor specificities of DPE2 and MalQ. As outlined above, DPE2 has an insertion in the GH77 catalytic domain and two N-terminal CBMs that are absent from MalQ (19). DPE2 also has a predicted coiled-coil motif between the CBM and GH77 domains (Fig. 8*A*), which is conserved in DPE2 from many organisms but absent from the chloroplastic 4-α-glucanotransferase DPE1 and from MalQ (Fig. 8*B*). Coiled-coiled motifs facilitate protein-protein interactions, in particular homodimer formation (31). Results from analyses of pure recombinant DPE2 by size exclusion chromatography, dynamic light scattering, and non-denaturing PAGE were all consistent with its existence as a homodimer (Figs. 2, *A* and *B*, and 8*C*). Previous research also suggested that DPE2 exists as a dimer or larger multimer in *Arabidopsis* and maize leaf extracts (32).

We attempted to discover the importance for DPE2 properties of the coiled-coil motif and the insertion in the GH77 domain by producing recombinant proteins in which these features were altered or missing. Proteins that lacked the coiled-coil motif or possessed a mutated form with an inserted β-turn motif predicted to ablate its function (33) were insoluble, suggesting that the motif is important for protein structural integrity. A DPE2 protein lacking the insertion in the GH77 domain was inactive and probably unfolded (from circular dichroism data; not shown). To establish whether the mechanism of action of DPE2 is the same as that of other GH77 enzymes despite the insertion, we established through mutagenesis that the enzyme requires for activity two of the three invariant residues that make up the catalytic triad of GH77 enzymes (Fig. 8*D*); a requirement for the third of these residues had been established previously (19). These data suggest that DPE2 has the same catalytic mechanism as other GH77 enzymes and thus that the amino acid insertion plays no major role in catalysis.

A previous study showed that the CBM20 domain of DPE2 is important for its actions on malto-oligosaccharides and its affinity for glucans. A mutant form of DPE2 lacking the N-terminal CBM20 had much higher disproportionation activity when supplied with maltose and short malto-oligosaccharides (Glc3 to Glc7) than native DPE2 and much lower activity when supplied with maltose and glycogen (19). Whereas the native enzyme bound to starch, the mutant did not. Steichen *et al.* (19) proposed that the CMB20 domain confers affinity for large glucans but also limits the use of small malto-oligosaccharides because it restricts the binding of substrate beyond subsite −1, as discussed above.

We extended these investigations by comparing the glucan-binding capacity of the CBM20s with that of native DPE2. Recombinant CMB20-1 (the N-terminal module) and native DPE2 both bound to starch and starch polymers, but CBM20-2 did not bind to these glucans (Fig. 8, *E* and *F*; purification shown in Fig. 2, *H* and *I*). These results suggest that the glucan-binding properties of DPE2 are largely conferred by CBM20-1. A similar conclusion was drawn by Steichen *et al.* (19), who reported that loss of CMB20-1 alone was sufficient to abolish the glucan binding properties of DPE2.

Given the influence of the CBM20 domain on the properties of DPE2, we investigated the impact on MalQ of the addition of this domain (containing both CBM20-1 and CBM20-2; purification shown in Fig. 2*G*). As expected, the domain conferred the ability to bind to starch and amylose (Fig. 8*G*). However, the fusion protein resembled MalQ rather than DPE2 with respect to its affinity for small malto-oligosaccharides. Using SPR, we found that the fusion protein had a very high affinity for Glc3 and Glc4 and progressively lower affinities for Glc5, Glc6, and Glc7 (Fig. 7). Although dissociation constants for the fusion protein were higher than for MalQ, they were at least 80 times lower than the dissociation constants of DPE2 for the same molecules.

The CMB20 domain did not confer on MalQ the ability to use carbohydrates other than glucans as acceptors. When incubated with the carbohydrate microarrays and [^14^C]maltose, the fusion protein labeled the same glucans as MalQ. It did not use any of the diverse carbohydrates used by DPE2, and it retained the ability to use the branched malto-oligosaccharides that were not used by DPE2 (Fig. 3*C*).

These results confirm that the major structural difference between DPE2 and MalQ, the presence of CBMs on DPE2, confers the capacity to bind to polyglucans. It also influences the affinity of the protein for small malto-oligosaccharides, but it does not confer the very low affinity for small malto-oligosaccharides seen in DPE2. Importantly, it does not confer the capacity to use heteroglycans. Thus, the use of heteroglycans as substrates must be attributable to some structural difference between MalQ and DPE2 other than the CBM20 domain. Future identification of this feature will be facilitated by our observation that the cytosolic glucan phosphorylase PHS2, which lacks obvious carbohydrate binding modules, can act on the same range of glycans as DPE2. It can also use the same glucosyl acceptor/donor sites on SHG_L_ as DPE2 (6). These data imply that some common structural feature of DPE2 and PHS2 is responsible for glycan binding.

##### Despite Their Different Properties, both DPE2 and MalQ Can Potentially Generate Glucosyl Buffers in Vivo

Our results lend weight to the current view that conversion of maltose to hexose phosphate in the cytosol at night uses the cytosolic heteroglycan as an intermediate. Although DPE2 has the general properties of a GH77 glucanotransferase, it is more complex than most GH77 enzymes, and it possesses a capacity to transfer glucosyl moieties from malto-oligosaccharides onto complex glycans that is thus far unique among GH77 enzymes. In addition, DPE2 cannot use small malto-oligosaccharides effectively as acceptor substrates; it requires a larger glucan or glycan as an acceptor. All of these features are consistent with the use of the cytosolic heteroglycan as an acceptor substrate by DPE2. Our results also show that MalQ could not directly substitute for DPE2 in the plant, because it lacks the capacity to transfer glucosyl moieties from maltose to cytosolic heteroglycan.

However, although MalQ could not replace the DPE2 function directly, it might be able to substitute for the proposed glucosyl buffer function of the pathway. Fettke *et al.* (11) suggested that in wild-type plants, cytosolic heteroglycan acts as a glucosyl “buffer” between maltose export from the chloroplast and hexose phosphate utilization in the cytosol, so that temporary fluctuations in one of these fluxes does not impact the other flux. Kartal *et al.* (20) provided a mechanism for this buffering action. They showed that DPE2 and PHS2 together can maintain polydispersity of glucosyl chains on cytosolic heteroglycan, in a system driven largely by entropy gradients. Comparison of the theoretical performance of this entropy-driven cytosolic heteroglycan pathway with an alternative hypothetical pathway in which maltose is metabolized to hexose phosphate without a polydisperse glucan intermediate showed that the former pathway can maintain a constant output of hexose phosphate in the face of fluctuations in maltose export from the chloroplast, but the latter, hypothetical pathway lacks this buffering capacity.

We reasoned that MalQ might provide a glucosyl buffer by disproportionating maltose to yield glucose and a polydisperse pool of malto-oligosaccharides. Glucose could be further metabolized by hexokinase, and malto-oligosaccharides could be further metabolized by cytosolic phosphorylase PHS2 (analogous to the metabolism of imported maltose in *E. coli*; see Fig. 1, *A* and *B*). We examined this possibility using the modeling approach described by Kartal *et al.* (20) (see “Experimental Procedures”).

**FIGURE 9.**
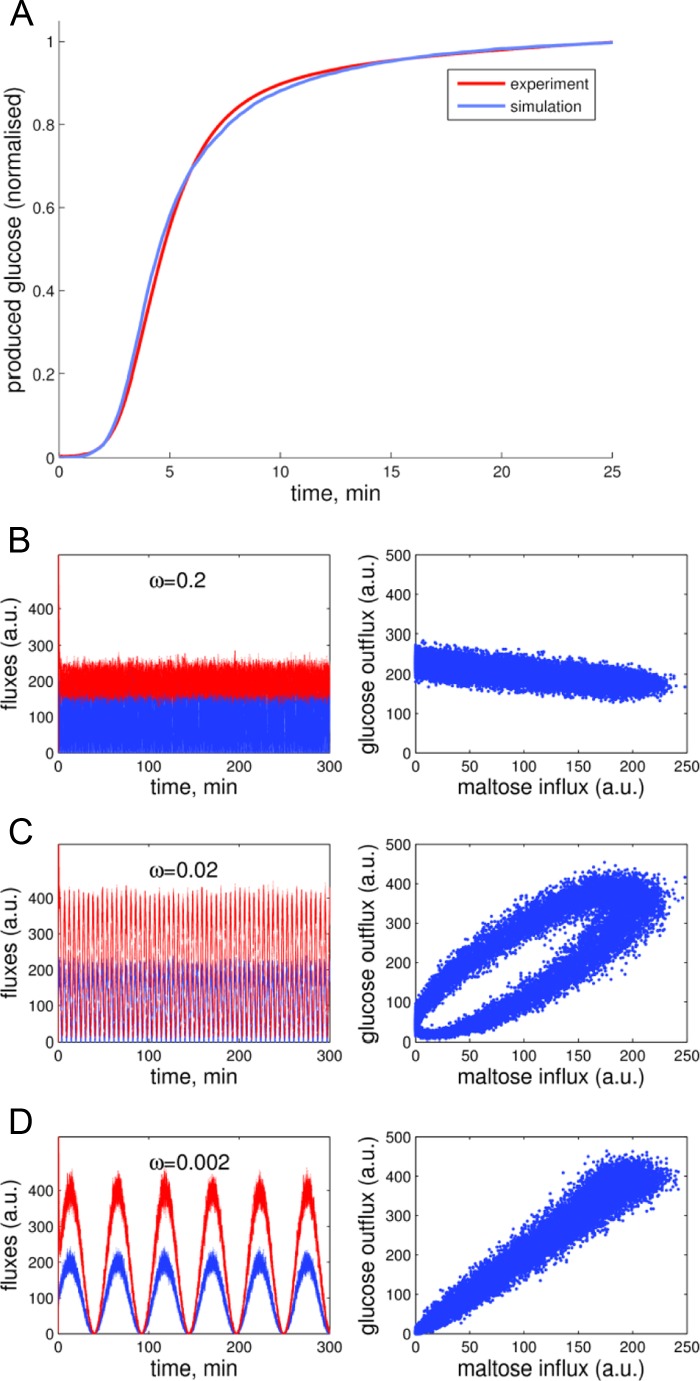
**Predictions of MalQ performance derived from modeling.** *A*, simulations of glucose production during incubation of MalQ with pure maltose. Experimentally observed glucose production (*red line*; data from Fig. 6) is compared with the simulated production of glucose (*blue line*) resulting from a mathematical model simulating identical conditions. To compare the simulation and experiment, the glucose produced is normalized to 1 after a reaction time of 25 min. *B–D*, the MalQ-mediated glucosyl buffer. Maltose influx was simulated with rapid (*B*), medium (*C*), or slow (*D*) fluctuations. The *left panels* show the maltose influx (*blue*) and glucose-phosphate outflux (*red*) rates as functions of time. The *right panels* show the relation between influx and outflux. It can be seen that rapid fluctuations are buffered efficiently (*B*, *right*), and the outflux rate is rather independent of the maltose influx. For slower fluctuations, the outflux follows the influx (*C* and *D*, *right*). *a.u.*, arbitrary units.

We first examined under which conditions MalQ can create a polydisperse pool of malto-oligosaccharides from pure maltose. Early work on MalQ (34) suggested that maltose cannot act as a glucosyl donor for this enzyme. Without further assumptions, this suggestion is not compatible with subsequent observations that the recombinant enzyme catalyzes disproportionation with pure maltose as initial substrate, in the absence of any added donor (16, 17) (experiments above). However, as discussed above, MalQ displays an extremely high affinity for short malto-oligosaccharides (dissociation constants of 0.26 and 0.41 μm for Glc3 and Glc4, respectively; Fig. 7). It can be assumed that both *in vivo* and *in vitro*, at least some MalQ proteins are bound to malto-oligosaccharides that can act as initial donors, allowing formation of free malto-oligosaccharides from maltose. These then act as donors and initiate the disproportionation reaction. With this assumption, the time course of glucose production observed from added maltose in *in vitro* assays with purified recombinant MalQ can readily be explained (Fig. 9*A*).

Once a polydisperse pool of malto-oligosaccharides is established, it can potentially act as a buffer between maltose influx and glucose-phosphate utilization, analogous to the cytosolic heteroglycan buffer (20). To explore the functioning of the malto-oligosaccharide buffer, we simulated a highly fluctuating maltose influx and a constant demand for glucose phosphate through a rapidly changing function representing the provision of maltose and a fixed rate constant for the glucose phosphate-removing reaction. Fig. 9*B* shows that a constant provision of glucose phosphate is maintained despite the highly variable maltose influx. An interesting property of the buffering mechanism is revealed by simulating slow fluctuations. The outflux of glucose phosphates follows the influx of maltose with a certain delay (Fig. 9*D*). This can be explained by the fact that any (slow) change in either supply or demand leads to an adaptation of the buffer size. As a consequence, such changes do not have an immediate effect on other processes but rather lead to a slow adaptation of the polydisperse buffer.

From these considerations, it seems likely that MalQ could bypass a requirement for DPE2 *in planta* if the primary function of the DPE2-cytosolic heteroglycan pathway is to provide a glucosyl buffer in the pathway of starch degradation. However, if DPE2 and/or cytosolic heteroglycan have other essential functions specific to their properties and structures, MalQ could not substitute for DPE2. We tested these ideas by introducing a construct encoding MalQ from *E. coli* into the *dpe2* mutant of *Arabidopsis* and examining the extent to which a wild-type phenotype was recovered in homozygous, MalQ-expressing transgenic lines.

##### Expression of MalQ Can Restore a Wild-type Phenotype in dpe2 Mutant Plants

**FIGURE 10.**
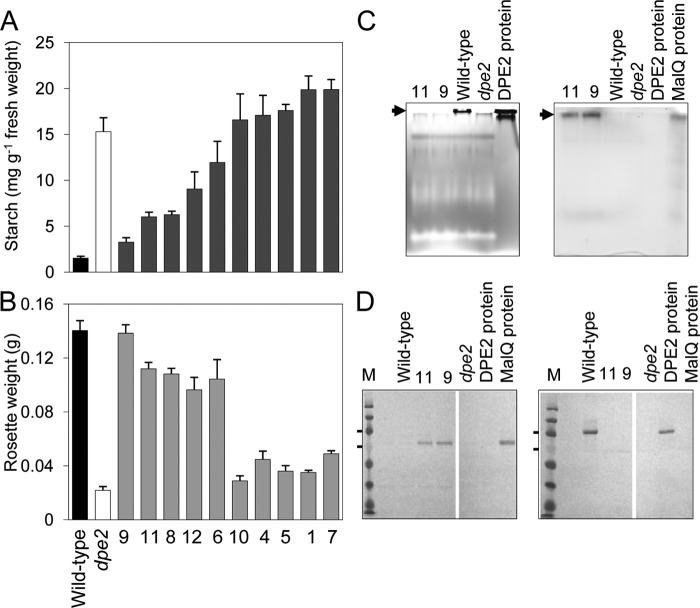
**Analysis of transgenic lines.** A construct encoding MalQ from *E. coli* was introduced into *dpe2* mutant plants, and independent homozygous transgenic lines were selected. *A* and *B*, starch content at the end of the night (*A*) and rosette fresh weight (*B*) for wild-type plants, *dpe2* mutant plants, and 10 transgenic lines. Plants were grown in 12-h light, 12-h dark and were harvested when 21 days old. Values are means of measurements on five individual plants for each line. *Error bars*, S.E. Experiments were also performed on *dpe2* plants transformed with the empty vector; results were the same as for *dpe2. C*, native gels developed for activity of DPE2 (*left*) and MalQ (*right*). Activity (*arrows*) in extracts of two transgenic lines (*dpe2* MalQ11 and *dpe2*MalQ9) is compared with that in wild-type and *dpe2* mutant plants and the activity of purified recombinant proteins. For plant extracts, each *lane* contains the same amount of protein. Recombinant MalQ shows no activity on DPE2 activity gels (not shown). *D*, immunoblots on nitrocellulose membranes of 9% SDS-polyacrylamide gels. Each *lane* contained either 60 μg of protein from a plant extract or 0.3 μg of purified recombinant DPE2 or MalQ protein. The *left blot* was developed with MalQ antiserum; the *right blot* was developed with DPE2 antiserum. *M*, molecular mass markers; the two markers indicated are 100 kDa (*top*) and 70 kDa (*bottom*).

*dpe2* mutant plants have up to 200 times more maltose than wild-type plants, elevated levels of starch, altered sugar metabolism, and reduced growth rates (3, 4). Among *dpe2* lines expressing MalQ, we found several with starch contents similar to wild-type rather than *dpe2* plants (Fig. 10*A*). End-of-night starch content was correlated with rosette fresh weight across the transgenic lines. The line with the lowest starch content (*dpe2*MalQ9) had the same fresh weight as wild-type plants, whereas lines with high starch contents had fresh weights comparable with *dpe2* (Fig. 10*B*). Two lines selected for further study had end-of-night starch contents of 3.3 (*dpe2*MalQ9) and 6.0 (*dpe2*MalQ11) mg of starch g^−1^ fresh weight, compared with wild-type and *dpe2* values of 1.5 and 15.3, respectively.

DPE2 and MalQ activities in extracts of the selected transgenic lines were analyzed by detection of iodine staining bands in glycogen-containing native gels incubated with maltose (for DPE2) or Glc7 (for MalQ) after electrophoresis (3). As expected, DPE2 activity was present in wild-type plants but absent from *dpe2* plants and the *dpe2*MalQ lines. MalQ activity was absent from wild-type and *dpe2* lines but present in the transgenic lines (Fig. 10*C*). Immunoblot analysis detected DPE2 protein in wild-type plants but not in *dpe2* or transgenic plants, and MalQ protein was detected only in transgenic plants (Fig. 7*D*; validation of antisera shown in Fig. 2*E*). Both native gel and immunoblot analyses indicated that the level of MalQ activity/protein was higher in *dpe2*MalQ9 than in *dpe2*MalQ11.

**FIGURE 11.**
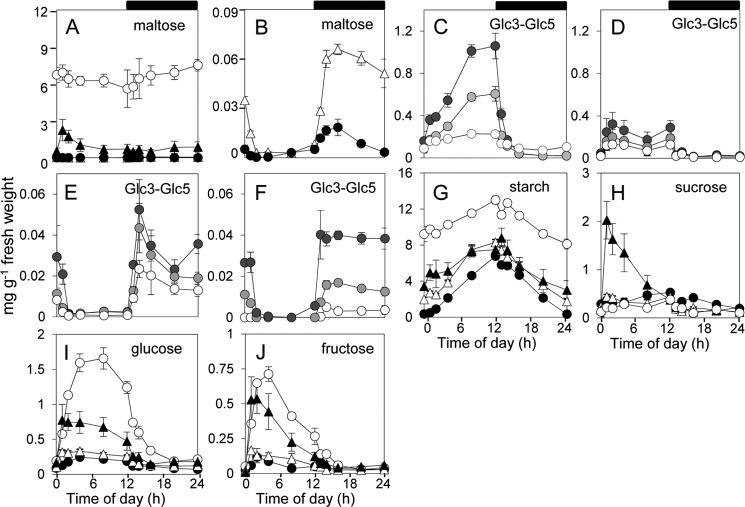
**Metabolite contents of transgenic lines.** Measurements were made on whole rosettes of 21-day-old plants grown in 12-h light, 12-h dark over a single day-night cycle. Values are means of measurements on five individual plants for each line. *Error bars*, S.E. *A*, maltose levels in *dpe2* mutant (*open circles*), *dpe2*MalQ11 (*closed triangles*), *dpe2*MalQ9 (*open triangles*), and wild-type (*closed circles*) plants. *B*, data for wild-type and *dpe2*MalQ9 plants from *A*, on an expanded scale. *C–F*, Glc3 (*dark gray*), Glc4 (*light gray*), and Glc5 (*white*) levels in *dpe2* mutant (*C*), *dpe2*MalQ11 (*D*), wild-type (*E*), and *dpe2*MalQ9 (*F*) plants. Note the different *y* axis scales. *G–J*, starch, sucrose, glucose, and fructose levels in *dpe2* mutant (*open circles*), *dpe2*MalQ11 (*closed triangles*), *dpe2*MalQ9 (*open triangles*), and wild-type (*closed circles*) plants.

Expression of MalQ strongly reduced the levels of maltose in *dpe2* mutant plants. In *dpe2*MalQ9, levels were at least 100-fold lower than in the *dpe2* mutant, and the daily pattern of change was similar to that of wild-type plants. In *dpe2*MalQ11, maltose was at least 10-fold higher than in wild-type plants but about 8-fold lower than in the *dpe2* mutant (Fig. 11, *A* and *B*). Levels of Glc3, Glc4, and Glc5 were similar in *dpe2*MalQ9 and wild-type plants and much lower than in *dpe2* plants. Levels rose at night and fell during the day in both genotypes. In *dpe2*MalQ11 plants, levels of these compounds were higher than in wild-type plants, and like those of *dpe2* plants, they rose during the day and fell at night (Fig. 11, *C–F*). Levels of starch and sugars were similar in *dpe2*MalQ9 plants and wild-type plants, whereas levels in *dpe2*MalQ11 plants were more similar to those of *dpe2* plants (Fig. 11, *G–J*).

**FIGURE 12.**
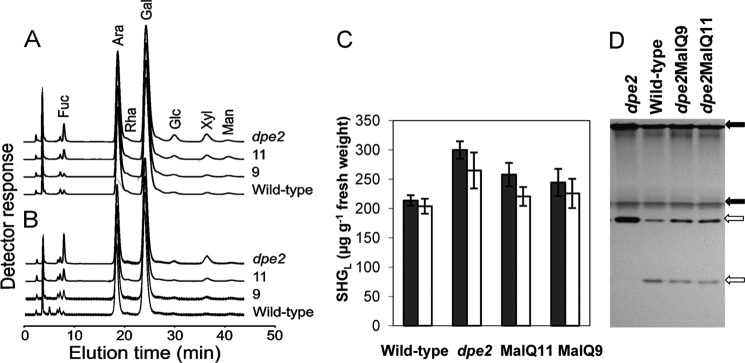
**Effects of MalQ expression on SHG_L_ and PHS2 in transgenic lines.** SHG_L_ was purified from mature leaves of *dpe2* mutant and wild-type plants and transgenic lines *dpe2*MalQ11 and *dpe2*MalQ9. The sugar composition of hydrolyzed SHG_L_ was analyzed by HPAEC-PAD. *A* and *B*, plants harvested at the end of the night (*A*) and the end of the day (*B*). *Fuc*, fucose; *Ara*, arabinose; *Rha*, rhamnose; *Gal*, galactose; *Glc*, glucose; *Xyl*, xylose; *Man*, mannose. *C*, amounts of SHG_L_ in mature leaves at the end of the day (*white*) and the end of the night (*black*). Values are means of three biological replicates. *Error bars*, S.D. *D*, phosphorylase activity in transgenic lines. Extracts containing 10 μg of protein were subjected to discontinuous native PAGE and developed for phosphorylase activity (9). *Closed arrows*, PHS2 (cytosolic) activity; *open arrows*, PHS1 (plastidial) activity (see Refs. 13, 41, and 42).

We reported previously that the *dpe2* mutation changes the composition of leaf cytosolic heteroglycans; the contents of xylose, mannose, and in particular glucose are higher in relation to arabinose and galactose than in wild-type plants (6). Expression of MalQ in *dpe2* plants reversed these changes. The composition of SHG_L_ from *dpe2*MalQ plants with high levels of MalQ resembled that of wild-type rather than *dpe2* plants (Fig. 12, *A* and *B*). *dpe2* plants contained more SHG_L_ than wild-type plants, and *dpe2*MalQ plants contained amounts intermediate between those of *dpe2* and wild-type plants (Fig. 12*C*).

Loss of *dpe2* increases glucan phosphorylase activity in *Arabidopsis* leaves (3). Using a native gel assay, we found that activities of both plastidial (PHS1) and cytosolic (PHS2) isoforms of phosphorylase were higher in *dpe2* than in wild-type extracts but were restored to nearly wild-type levels in both *dpe2*MalQ lines (Fig. 12*D*).

**FIGURE 13.**
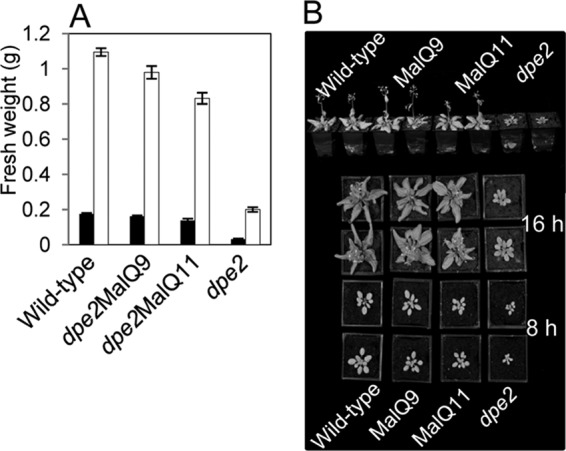
**Growth of wild-type and transgenic plants.** *A*, fresh weights of wild-type, *dpe2*, and *dpe2*MalQ lines grown in 16-h light, 8-h dark (*white*) or 8-h light, 16-h dark (*black*) for 28 days. Values are means of measurements on 10 plants for each line. *Error bars*, S.E. *B*, plants from the experiment in *A*. Two plants of each line are shown. *Top*, side view of plants grown with 16-h light, 8-h dark. *Bottom*, plants grown in either 16-h light or 8-h light, as indicated.

Our results thus far indicated that MalQ can largely replace the functions of DPE2/cytosolic heteroglycan, allowing nearly normal plant metabolism and growth in 12-h light, 12-h dark cycles. However, shortfalls in the capacity of MalQ to replace the endogenous pathway might be more obvious under long night conditions, in which plant growth is strongly dependent on optimal conversion of starch to hexose phosphates. Accordingly, we grew *dpe2*MalQ plants at a range of day lengths. Day length had no obvious effect on the relationship between the fresh weights of wild-type and *dpe2*MalQ plants. In batches of 28-day-old plants grown with 18-, 16-, and 8-h night periods, *dpe2*MalQ9 plants had 108, 89, and 92% of the fresh weight of wild-type plants, respectively (see also the independent experiment in Fig. 13). Thus, MalQ can effectively replace the functions of DPE2 and the cytosolic heteroglycan even in conditions in which growth is highly dependent on this pathway.

##### Possible Evolutionary Origins of the DPE2-Cytosolic Heteroglycan Pathway

Our results indicate that DPE2 and the cytosolic heteroglycan have no specific structural or functional properties that are essential for the conversion of maltose to hexose phosphate (or indeed other aspects of cellular metabolism) *in planta*. This conclusion arises because, under the range of growth conditions we examined, DPE2/heteroglycan can be replaced by MalQ without obvious detrimental effects on plant growth.

This finding is surprising in light of the genetic and biochemical complexity of the heteroglycan (its synthesis must require numerous enzymes of sugar interconversion and polymerization) and the high level of conservation of the pathway among photosynthetic organisms. Phylogenetic analyses by Ball and colleagues (35–39) offer a possible evolutionary explanation for this conundrum. These authors propose a complex evolutionary origin of chloroplastic starch metabolism, in which pathways of glucan metabolism derived from the eukaryotic host and the prokaryotic symbiont have been “rewired” by modification, duplication, and subcellular relocation. Their analyses indicate that DPE2 was acquired by the Archaeplastida from their eukaryotic ancestor, in which it may have been involved in maltose metabolism associated with cytoplasmic α-glucan turnover. In this ancestral organism, the acceptor substrate for DPE2 would have been an α-glucan rather than heteroglycan, and the CBMs of DPE2 may have had a role in determining its affinity for α-glucan. During the evolution of the Chloroplastida, it is proposed that α-glucan turnover relocated to the plastid. Evolution of the plastid envelope maltose transporter MEX1 (found only in the Chloroplastida) (36) meant that cytosolic DPE2 continued to provide the route for metabolism of maltose produced by starch degradation but no longer had access to α-glucan as an acceptor substrate. Presumably, the use of cytosolic heteroglycan as an acceptor substrate arose at this point. DPE2 either already possessed the capacity to use glycans as substrates or acquired features that enhanced affinity for existing cytosolic heteroglycans in response to relocation of α-glucan metabolism to the plastid.

As a parallel evolutionary scenario, we raise the possibility that the cytosolic heteroglycan might reflect a form in which the eukaryotic host initially acquired carbon from the photosynthetic symbiont. Ball *et al.* (38) have argued persuasively that the main form in which carbon was exported from the cyanobacterial symbiont was ADP-glucose, fuelling an existing pathway of α-glucan synthesis in the cytosol of the host cell. We suggest that the cyanobacterial symbiont may also have continued to export the glycans that would have constituted its exopolysaccharides in the free-living state. Many modern cyanobacteria release exopolysaccharides with compositions similar to that of cytosolic heteroglycan; arabinose, xylose, galactose, fucose, rhamnose, and glucose are all common residues (40). Perhaps catabolism of these exopolysaccharides provided an important source of carbon for the host cell, and cytosolic heteroglycan is an evolutionary remnant of this first interface between photosynthetic and host cell metabolism.

## Footnotes

9 The abbreviations used are:

SHG: soluble heteroglycan
CBM: carbohydrate binding module
SPR: surface plasmon resonance
GH77: glycoside hydrolase family 77
TEV: tobacco etch virus
HPAEC: high performance anion exchange chromatography
PAD: pulsed amperometric detection
Tricine: *N*-[2-hydroxy-1,1-bis(hydroxymethyl)ethyl]glycine
IMAC: immobilized metal affinity chromatography.
